# Validation of the Non-Suicidal Self-Injury Scar Cognition Scale (NSSI-SCS) in a Russian-Speaking Sample

**DOI:** 10.11621/pir.2025.0408

**Published:** 2025-12-01

**Authors:** Alexandrina A. Grigoreva, Anastasia K. Kondratovich, Farah M. Trabelsi, Arina N. Mokritskaya, Maksim P. Marachev, Ilya A. Fedotov

**Affiliations:** a Sechenov First Moscow State Medical University, Moscow, Russia; b Ryazan State Medical University, Ryazan, Russia; c Neurocentre of medical-psychological correction and rehabilitation, Moscow, Russia

**Keywords:** self-injurious behavior, attitudes toward scars, consequences of self-injury, non-suicidal self-injury, cognitive assessment

## Abstract

**Background.:**

Non-suicidal self-injury (NSSI) scars carry significant emotional weight, but no validated tools existed to assess related cognitions in Russian-speaking clinical practice.

**Objective.:**

This study aimed to adapt and validate the Russian-language version of the Non-Suicidal Self-Injury Scar Cognition Scale (NSSI-SCS) and examine its psychometric properties in a clinical Russian-speaking sample.

**Design.:**

The study recruited 262 participants with NSSI history and visible scars from psychiatric hospitals. The adaptation used forward-back translation and cross-cultural adaptation. Reliability was assessed with Cronbach’s alpha and McDonald’s omega. Construct validity was evaluated via Confirmatory (CFA) and Exploratory Factor Analysis (EFA). Convergent and discriminant validity were examined through correlations with measures of suicidality, social anxiety, rumination, body image dysphoria, and NSSI functions.

**Results.:**

CFA indicated a poor fit for the original five-factor model. EFA supported a culturally adapted three-factor structure: ‘Hopelessness’ (suicidal ideation, weakness), ‘Shame’ (social stigmatization), and ‘Inner Strength’ (resilience). The 21-item scale demonstrated high internal consistency (α = .876 total). Convergent validity was conirmed by signiicant positive correlations of ‘Hopelessness’ and ‘Shame’ with suicidality, social anxiety, rumination, and body dysphoria. ‘Inner Strength’ showed discriminant validity through non-signiicant correlations with these clinical measures but positive correlations with NSSI functions.

**Conclusion.:**

The Russian NSSI-SCS is a valid and reliable tool. Its three-factor model provides a clinically useful framework for understanding the psychological impact of NSSI scars, facilitating targeted interventions and further research.

## Introduction

Non-suicidal self-injury (NSSI) is a prevalent form of self-directed aggression that remains a significant public health concern, prompting a substantial increase in research over recent decades (Duarte et al., 2020; Gardner et al., 2021). Epidemiological studies indicate that 17% to 36% of individuals in the general population engage in NSSI (Kiekens et al., 2023; Nock et al., 2006; Polskaya, 2017), with prevalence rates rising to 40-61% in clinical samples (Akselrov et al., 2021; Daryin & Zaitseva, 2023; Zinchuk et al., 2019). This behavior is associated with a wide spectrum of mental health problems, adversely affecting both immediate functioning and long-term prognosis (Akselrov et al., 2021; Nock et al., 2006).

The scarring that results from NSSI represents a significant physical and psychological consequence. These scars often have a more detrimental impact on body image than those from accidental injuries or surgeries (Dyer et al., 2013; Ho et al., 2018). For many, scars act as potent triggers, reactivating the negative memories and psychological distress that precipitated the self-injurious act (Bachtelle & Pepper, 2015; Burke et al., 2017). Research indicates that scars carry a distinct emotional significance; for instance, one study found that 65% of individuals with NSSI perceive their scars negatively, associating them with personal failures and regrets (Foley, 2022). Negative experiences such as social stigmatization, shame, guilt, and regret oten drive individuals to seek surgical or cosmetic treatments to enhance the social acceptability of their scars (Edriss et al., 2022; Ho et al., 2018; Linington, 2024; Takaya et al., 2020). Conversely, a parallel body of evidence suggests that some individuals perceive their self-injury scars positively, viewing them as marks of personal strength and survival (Kendall et al., 2021; Linington, 2024). This interpretation of scars can have a positive efect on psychotherapeutic work.

Consequently, the cognitive appraisal of NSSI scars is a complex and multifaceted phenomenon that necessitates reliable assessment tools. To address this gap, Burke et al. (2017) developed the Non-Suicidal Self-Injury Scar Cognition Scale (NSSI-SCS), The original English version of the NSSI-SCS is a 26-item self-report questionnaire that assesses scar-related cognitions over the preceding two weeks. It measures five distinct types of appraisals: (1) Reminder Cognitions (scars reactivating memories and emotions linked to the self-injury), (2) Social Cognitions (scars as a source of social stigma and judgment), (3) Weak Cognitions (scars associated with shame, unattractiveness, and weakness), (4) Suicide Cognitions (scars provoking suicidal thoughts), and (5) Positive Cognitions (scars viewed as symbols of resilience and overcoming hardship). At the time of our study, the scale had no validated translations and was available only in English.

While several psychometric instruments available in Russian research practice address various aspects of NSSI — such as the experiences during the act itself (*e.g.*, ISAS I; Kibitov et al., 2025), its functions (*e.g.*, ISAS II; Kibitov et al., 2025; Zinchuk et al., 2023), motivations (e.g., Scale of Reasons for Self-Injurious Behavior; Polskaya, 2014), and the severity of harm (e.g., The Deliberate Self-Harm Inventory; Kuznetso-va et al., 2021) — none specifically target the cognitive and emotional consequences of the resulting scars. Therefore, validating the NSSI-SCS within the Russian cultural context is crucial to address this methodological gap. A culturally adapted version would provide researchers and clinicians with a vital tool for assessing attitudes toward NSSI scars, which is essential for understanding their long-term consequences and developing targeted interventions. The aim of the present study was to adapt and validate the Russian-language version of the NSSI-SCS in a sample of individuals with a history of non-suicidal self-injury.

### NSSI-SCS overview

The systematic study of cognitive appraisals of non-suicidal self-injury (NSSI) scars was preceded by qualitative explorations and preliminary quantitative evaluations attempts. Early conceptualization efforts include the work of Lewis & Mehrabkhani (2016), who conducted a thematic analysis of online forum discussions where individuals shared experiences with self-injury scars. They identified key themes in scar appraisal, including shame, embarrassment, reminders of past pain, and the perception of scars as symbols of survival and strength. These themes laid the groundwork for future conceptual domains of the NSSI-SCS. Among the first attempts to create a quantitative scale were the studies by Bachtelle & Pepper (2015), who developed measures for shame and personal growth related to scars. However, their instruments were limited by a small number of items, the absence of factor analysis for validation, and a dichotomous response format, which prevented a full exploration of the complex experiences associated with scarring. Another instrument, the Non-Suicidal Self-Injury Assessment Tool (NSSI-AT) by Whitlock et al. (2014), included items on scars, but the reliability of the relevant subscale was low. Th us, by 2017, while a qualitative understanding of the phenomenon existed, quantitative measurement attempts were fragmented and unreliable. Th ere was no unified, psycho-metrically sound tool to comprehensively assess the diverse cognitions about scars. The Burke et al. (2017) NSSI-SCS systematizing previous findings into a single, valid scale.

The original English version of the NSSI-SCS was designed as a comprehensive self-report instrument to assess the wide spectrum of thoughts and beliefs individuals associate with their NSSI scars. Item development was based on previous research, early scale development attempts, and existing theoretical models concerning NSSI, suicidal behavior, and psychotrauma. The scale’s core premise is that NSSI scars are not merely physical remnants but potent stimuli imbued with signiicant psychological meaning, which can influence ongoing distress, self-perception, and clinical outcomes. Crucially, understanding these appraisals is essential for identifying potential therapeutic targets for patients with diferent experiences in connection with NSSI.

Initially, Burke generated a 26-item questionnaire distributed across four hypothesized domains: Reminder Cognitions, Social Cognitions, Suicide Cognitions, and Positive Cognitions. A fifth domain, Weak Cognitions, was not initially planned but emerged from empirical data. An Exploratory Factor Analysis (EFA) on a sample of 110 students with NSSI scars revealed this distinct factor, which was subsequently interpreted post-hoc. Consequently, the i nal ive-factor model (Reminder, Social, Suicide, Positive, Weak) resulted from a combination of theoretical groundwork, qualitative data, and empirical validation.

SCS-Reminder Cognitions (Items 3, 4, 9, 15). This subscale assesses the extent to which an individual’s NSSI scars serve as persistent and intrusive reminders of past negative experiences, failures, and stressful life events. Conceptually, the scars are appraised as cues that trigger aversive memories related to the circumstances that originally prompted the self-injurious behavior. This is grounded in trauma theory, which posits that permanent physical markers can act as constant retrievers of painful memories, thereby potentially maintaining or exacerbating psychological distress (Weaver et al., 2007). Representative Items: “My scar(s) bring back memories of things that I don’t want to remember,” “My scar(s) remind me of stressful things that happened to me in the past.”

SCS-Social Cognitions (Items 8, 10, 16, 18, 23). This subscale measures cognitions related to the perceived social stigma and interpersonal consequences of having visible NSSI scars. It captures a dual-layered experience: (1) the external component of perceived stigma, including the belief that others judge, stare, or are embarrassed by one’s scars, and (2) the internalized component, where this anticipated social judgment fuels profound feelings of personal shame. This dimension is supported by literature on the stigma associated with mental illness, where scars are oten perceived as physical manifestations of a disorder (Hodgson, 2004; Lewis & Mehrabkhani, 2015). Representative Items: “My scar(s) make me embarrassed in front of other people,” “I think that people judge me because of my scar(s).”

SCS-Suicide Cognitions (Items 14, 17, 19, 22, 25, 26). This subscale measures the cognitive linkage between NSSI scars and suicidal ideation and capability. It encompasses two key components derived from the Interpersonal-Psychological Theory of Suicide (IPTS; Van Orden, 2008): (a) scars fostering feelings of hopelessness and entrapment, which fuel suicidal desire, and (b) scars contributing to an acquired capability for suicide by reducing the fear of death and increasing the perceived competence to enact lethal self-harm. Representative Items: “My scar(s) make me feel hopeless,” “My scar(s) make me feel less afraid of dying.”

SCS-Positive Cognitions (Items 1, 2, 5, 11, 20). In contrast to the negative domains, this subscale captures positively valenced interpretations of NSSI scars, measuring appraisals of scars as symbols of personal strength, resilience, and survival. Cognitions here include viewing scars as a source of pride, hope, and evidence of having overcome significant adversity (Bachtelle & Pepper, 2015; Kendall et al., 2021; Lewis & Mehrabkhani, 2015). Representative Items: “My scar(s) represent how strong I am emotionally,” “My scar(s) represent how strong I am physically.”

SCS-Weak Cognitions (Items 6, 7, 12). This subscale reflects a self-perception of inherent weakness and vulnerability intrinsically linked to the scars. While Reminder Cognitions focus on the scars’ function as triggers for external memories of past events, Weak Cognitions are directed inward, representing a global, negative self evaluation in the present. They encompass feelings of fear and the belief that the scars are a testament to one’s core inadequacy and inability to cope. Representative Items: “My scar(s) remind me that I am weak,” “My scar(s) make me feel afraid.”

In summary, the NSSI-SCS provides a nuanced, multidimensional framework for understanding how individuals cognitively process their self-injury scars. Its five sub-scales illuminate the complex and often contradictory ways scars can be perceived — as sources of social shame, painful reminders, self-perceived weakness, catalysts for suicidal thinking, and, importantly, personal strength. This comprehensive assessment offers a critical tool for both research into the long-term psychological impact of NSSI, which makes it possible to use this information in formulating a clinical prognosis and choosing a method of influencing symptoms.

## Methods

### Participants

The study sample comprised outpatient and inpatient individuals aged 18 to 70 who sought specialized psychiatric care and presented with at least one visible NSSI-relat-ed scar, as confirmed by self-report or corroboration from relatives. Participants were recruited via a consecutive sampling method from the clinical departments of the N.N. Bazhenov Regional Clinical Psychiatric Hospital (Ryazan) and the private outpatient neuropsychiatric clinic “Neurocenter” (Moscow) between November 2023 and January 2025. The sample size was determined a priori based on the psychometric standard of 10 participants per scale item (Nunnally, 1978).

Inclusion Criteria were: 1) age 18-70 years; 2) presence of at least one scar confirmed to result from NSSI; 3) a primary ICD-10 diagnosis of Mood (Affective) Disorders (F30-F39), Neurotic, Stress-related, and Somatoform Disorders (F40-F48), Behavioural Syndromes associated with Physiological Disturbances (F50-F59), or Disorders of Adult Personality and Behaviour (F60-F69); and 4) provision of written informed consent.

Exclusion Criteria included: 1) a documented history of Organic Mental Disorders (F00-F09), Substance Use Disorders (F10-F19), Schizophrenia Spectrum Disorders (F20-F29), or Intellectual Disabilities (F70-F79); and 2) a current psychotic episode, acute intoxication, catatonia, or signiicant cognitive impairment as assessed through clinical examination and medical record review.

The initial screening identified 329 eligible individuals aged 18 to 53 years (M = 23.3, SD = 5.385). From this cohort, 262 respondents who provided complete datasets and fully met the inclusion criteria constituted the inal analytical sample.

The final sample was predominantly female (74.4%), with 19.8% male and 5.7% not reporting gender. The most frequent educational attainment was incomplete higher education (30.5%), followed by secondary vocational (26.3%) and higher education (21.7%). Most participants were single (52.7%), and 34.3% were employed at the time of the assessment (see *Table 1* for detailed sociodemographic and clinical characteristics).

**Table 1:** Clinical and Socio demographic Characteristics of the Sample

Characteristics	M (SD)\N (%)
Age	23.5 (5.385)
Gender	
Male	52 (19.8%)
Female	195 (74.4%)
Not reported	15 (5.7%)
Education	
Primary school education	13 (4.9%)
Secondary school education	43 (16.4%)
Vocational secondary education	69 (26.3%)
Higher education	57 (21.7%)
Incomplete higher education	80 (30.5%)
Occupation	
Employed	90 (34.3%)
Studying	76 (29%)
Employed and studying	46 (17.5%)
Unemployed and not studying	50 (19.1%)
Marital status	
Married	28 (10.7%)
Single	138 (52.7%)
Cohabitation	45 (17.2%)
In a relationship (not cohabiting)	45 (17.2%)
Divorced	6 (2.3%)
Psychiatric diagnosis	
F40-48 Neurotic, Stress-related, and Somatoform Disorders	142 (54.2%)
F60-69 Disorders of Adult Personality and Behaviour	21 (8%)
F30-39 Mood [Affective] Disorders	99 (37.8%)

The majority of participants (54.2%) were diagnosed with disorders from ICD-10 block F40-F48 (Neurotic, stress-related, and somatoform disorders). Afective disorders (F30-F39) constituted the second most prevalent diagnostic category (37.8%), while disorders of adult personality and behaviour (F60-F69) were less frequent (8%).

### Procedure

The adaptation and validation process commenced with obtaining official authorization from the original author of the NSSI-SCS, Professor Taylor Burke. A comprehensive validation protocol was subsequently implemented, comprising the following sequential stages: (a) forward-back-translation and cross-cultural adaptation; (b) assessment of sampling adequacy and evaluation of internal consistency reliability; (c) confirmatory factor analysis (CFA) to test the original model’s fit; (d) exploratory factor analysis (EFA) using Principal Component Analysis with Oblimin rotation to investigate the underlying factor structure; and (e) examination of convergent and discriminant validity through correlation analyses with established psychological measures.

The translation and cross-cultural adaptation of the NSSI-SCS items were conducted by a panel of three bilingual experts (two psychiatrists and one clinical psychologist). The expert group reviewed and approved the final Russian version after reconciling the back-translated text with the original. This version was piloted on a sample of 30 individuals with a history of NSSI; no participants reported difficulties in comprehending the instructions or item content.

#### Data Collection

Data were collected in a single session. Eligible participants, identiied through consecutive sampling, received full disclosure of the study’s purposes and provided written informed consent. The testing procedure began with a sociodemographic and clinical questionnaire, which included a critical screening item: “Do you have any scars from self-injury?” Response options were: 1) Yes, in visible areas; 2) Yes, in concealed areas; 3) No. This item, informed by Burke et al. (2017) on the clinical relevance of scar visibility, screened out individuals without scars and documented scar location. Participants then completed a battery of six self-report measures, selected for their theoretical alignment with the cognitive appraisals measured by the NSSI-SCS.

#### Instruments of Measurement

1. *Non-Suicidal Self-Injury Scar Cognition Scale (NSSI-SCS)*. The NSSI-SCS is designed to assess cognitive and emotional responses to self-injury scars. The 26-item scale measures five domains of scar appraisal: Reminder Cognitions, Social Cognitions, Weak Cognitions, Suicide Cognitions, and Positive Cognitions. Respondents rate items on a 5-point Likert scale from 1 (“Not at all”) to 5 (“Extremely”).
2. *Ruminative Responses Scale (RRS;* Pugovkina et al., 2021). This scale assesses a ruminative cognitive style, characterized by a passive, repetitive focus on negative emotions. It was selected to evaluate convergent validity with the “Reminder Cognitions” subscale, based on the premise that scars acting as reminders of distress would correlate with a general tendency toward rumination.
3. *Liebowitz Social Anxiety Scale* (LSAS; Grigorieva & Enikolopov, 2016). The LSAS measures social anxiety through fear and avoidance subscales. It was paired with the “Social Cognitions” subscale, as cognitions of social stigmatization and embarrassment related to scars are a direct manifestation of social anxiety.
4. *Inventory of Statements about Self-Injury (ISAS-II;* Zinchuk et al., 2023). The ISAS-II assesses interpersonal and intrapersonal functions of NSSI. It was used to examine the relationship with the “Positive Cognitions” subscale, hypothesizing that appraising scars as a symbol of inner strength would be linked to specific self-injury functions (e.g., affect regulation, autonomy).
5. *Situational Inventory of Body-Image Dysphoria (SIBID;* Baranskaya et al., 2008). The SIBID measures context-specific body-image distress. It was aligned with the “Weak Cognitions” subscale, as cognitions of physical fl aw and weakness associated with scars were expected to correlate with general body-image dys-phoria.
6. *Beck Scale for Suicide Ideation (BSSI;* Beck et al., 1979). This scale quantifies the intensity of suicidal intent. It was correlated with the “Suicide Cognitions” sub-scale, testing the critical hypothesis that scar-related suicidal ideation would show a strong association with a standardized measure of suicidality.

In the present study, Cronbach’s alpha and McDonald’s Omega Coefficients for each tool were also calculated (see *Table 2*).

**Table 2:** Internal Consistency Reliability of the Study Measures: Cronbach’s Alpha and McDonald’s Omega Coefficients

Scales	Cronbach’s α	McDonald’s ω
Liebowitz Social Anxiety Scale	.900	.920
Inventory of Statements about Self-Injury, ISAS-II	.781	.798
Ruminative Responses Scale	.803	.858
Beck Scale for Suicide Ideation	.875	.877
Situational Inventory of Body-Image Dysphoria	.956	.957

Furthermore, a supplemental item evaluating scar location was included in the assessment protocol. This inclusion is clinically justified, as the visibility of scars can exacerbate self- and social stigma and may act as a situational trigger for maladaptive self-regulation by reactivating distressing memories (Burke et al., 2017).

#### Data Analysis

Statistical analyses were performed using Microsoft Excel 2013 and Jamovi version 2.3.21. Prior to conducting the main analyses, the normality of the data distribution was assessed using the Shapiro-Wilk test. Since the data significantly deviated from a normal distribution, non-parametric statistical methods were employed for all subsequent analyses.

The suitability of the data for factor analysis was evaluated using the Kaiser-Meyer-Olkin (KMO) measure of sampling adequacy and Bartlett’s test of sphericity. The high KMO value and the signiicant result of Bartlett’s test conirmed the adequacy of the sample and the presence of sufficient inter-item correlations to proceed with factor analysis.

To examine the factorial structure of the questionnaire, both Exploratory Factor Analysis (EFA) and Confirmatory Factor Analysis (CFA) were conducted. Principal Component Analysis (PCA) with Oblimin rotation was used for the EFA. Items were retained if they exhibited a minimum factor loading of .5 on their primary factor.

The CFA was performed to verify the original five-factor model proposed by the scale’s authors. Model fit was assessed using multiple indices: the Chi-square statistic (χ^2^), the Comparative Fit Index (CFI), the Tucker-Lewis Index (TLI), the Root Mean Square Error of Approximation (RMSEA) with its 90% conidence interval, and the Standardized Root Mean Square Residual (SRMR). Standard thresholds for acceptable model fit were applied (CFI/TLI > .90, RMSEA < .08, SRMR < .08). Th e Chi-Square Diference Test was used for direct model comparison.

The internal consistency reliability of the scale and its subscales was evaluated using both Cronbach’s (α) and McDonald’s (ω). Convergent and discriminant validity were assessed by examining bivariate correlations between the NSSI-SCS sub-scales and scores from established external measures, including the Beck Scale for Suicide Ideation (BSSI), the Liebowitz Social Anxiety Scale (LSAS), the Ruminative Responses Scale (RRS), and the Situational Inventory of Body-Image Dysphoria (SI-BID). Spearman’s rank correlation coefficient (ρ, rho) was used for these analyses due to the non-normal data distribution.

To investigate diferences in NSSI-SCS scores based on scar location (visible vs. hidden), the non-parametric Mann-Whitney U test was employed. For all statistical tests, a two-tailed p-value p<.05 was considered statistically significant.

In addition to the correlation analyses, a quartile comparison was conducted to further examine the scale’s validity and its ability to discriminate between respondents with polarized scores. Participants scoring in the highest (Q4) and lowest (Q1) quartiles on each NSSI-SCS subscale were compared using the Mann-Whitney U test. This analysis serves to complement the correlation analysis by not only establishing linear relationships but also quantifying the magnitude of differences between groups with minimal versus maximal levels of scar-related cognitions across key clinical and functional variables. This approach provides robust evidence for the discriminative validity of the subscales.

## Results

### Internal Consistency and Structural Validity

At the initial stage of statistical analysis, the suitability of the data for factor analysis was assessed. The Kaiser-Meyer-Olkin measure of sampling adequacy yielded a value of .866, and Bartlett’s test of sphericity was statistically significant (χ2 = 2691, df = 210, p < .001). Th ese results confirmed that the data contained a sufficient number of meaningful intercorrelations to proceed with factor analysis. To verify the original five-factor structure of the NSSI-SCS in the Russian-speaking clinical sample, a confirmatory factor analysis (CFA) was conducted (see *Table 3*).

**Table 3:** Model Fit Indices for the Confirmatory Factor Analysis (CFA) of the Original NSSI-SCS Five-Factor Structure

Factor	NSSI-SCS Statement	Factor Loading	SE	Z	ρ	Std. Loading
1 Reminder Cognitions	3. My scars bring back memories of things that I don’t want to remember	.8012	.085	9.394	< .001	.6072
	9. My scars remind me of stressful things that happened to me in the past	.7729	.081	9.507	< .001	.605
	15. My scars make me think about my failures and mistakes.	1.0333	.073	14.22	< .001	.8205
	4. My scars make me think about how weak I used to be	.7976	.087	9.125	< .001	.5944
2. Social Cognitions	10. My scars make me embarrassed in front of other people.	1.1282	.073	15.47	< .001	.8506
	23. I think that people judge me because of my scars.	1.0856	.081	13.34	< .001	.769
	16. I think that people stare at my scars.	.8331	.078	10.71	< .001	.6544
	18. My scars make me feel unattractive.	.9049	.072	12.56	< .001	.7359
	8. My scars make me feel shame.	.9427	.08	11.84	< .001	.7064
3. Positive Cognitions	1. My scar(s) represent how strong I am emotionally.	.7956	.064	12.51	< .001	.7577
	20. My scars make me feel hopeful about my future.	.564	.054	10.43	< .001	.6535
	2. My scar(s) represent how strong I am physically.	.4667	.059	7.955	< .001	.5317
	11. My scars make me feel tough, like I can get through anything.	.9671	.064	15.06	< .001	.8651
	5. My scars make me feel proud that I got through a very tough time	.9132	.078	11.67	< .001	.7094
4. Weak Cognitions	6. My scar(s) remind me that I am weak.	1.1533	.081	14.32	< .001	.8237
	7.My scars make me feel afraid;	.4434	.068	6.549	< .001	.4468
	12. My scars make me feel like I am weak	.9789	.07	14.02	< .001	.8085
5. Suicide Cognitions	26. My scars make me feel less afraid of dying.	.4863	.08	6.08	< .001	.4096
	22. My scars make me feel like I can’t change anything, like I’m stuck.	1.0915	.08	13.6	< .001	.7818
	17. My scars make me feel like I want to kill myself.	.5545	.055	10.02	< .001	.6236
	14. My scars make me feel hopeless.	.9206	.063	14.59	< .001	.8232
	25. I think that my scars make me unique.	-.016	.076	-.203	.839	-.0144
	19. My scars make me feel like I could kill myself if I wanted to.	.9394	.09	10.42	< .001	.6505

Analysis of the factor loadings revealed considerable variability across the different factors. The items of Factor 2 (“Social Cognitions”) demonstrated the strongest diagnostic utility, with standardized loadings ranging from .654 to .851. Factor 1 (“Reminder Cognitions”) and Factor 3 (“Positive Cognitions”) also showed adequate loadings for the majority of their items (.532-.820 and .532-.865, respectively).

However, several problematic aspects of the five-factor model were identi-ied. Factor 4 (“Weak Cognitions”) included Item 7 with a low standardized loading (.447). Factor 5 (“Suicide Cognitions”) contained a statistically non-signiicant Item 25 (p = .839) with a minimal loading (-.014). Several items demonstrated loadings below the recommended threshold of .50 (Items 2, 7, 26). The considerable variability in loadings within the factors indicates heterogeneity of the measured constructs.

The unsatisfactory nature of the initial model is further supported by the values of key goodness-of-fit indices: χ^2^(220) = 817, p < .001; CFI = .781; TLI = .748; RMSEA = .107 (90% CI: .099-.115); SRMR = .100 (see *Table 4*). All these indices failed to meet the conventional thresholds indicative of a good model fit (CFI/TLI > .90; RMSEA < .08; SRMR < .08).

**Table 4:** Goodness-of-Fit Indices for the Five-Factor NSSI-SCS Model

χ^2^ (Chi-square)		df (Degrees of freedom)		p-value	
817		220		< .001	
				RMSEA 90% CI	
**CFI**	**TLI**	**SRMR**	**RMSEA**	**Lower Bound**	**Upper Bound**
.781	.748	.1	.107	.0994	.115

Analysis of the internal reliability of the ive-factor NSSI-SCS structure yielded satisfactory results for the factors “Weak Cognitions” (α = .702) and “Suicide-Related Cognitions” (α = .741), which met the minimum acceptable thresholds (see *Table 5*).

**Table 5:** Internal Reliability Indices for the Five-Factor NSSI-SCS Structure (Cronbach’s Alpha, McDonald’s Omega)

Factor	Cronbach’s α	McDonald’s ω
1. Reminder Cognitions	.774	.775
2. Social Cognitions	.858	.861
3. Positive Cognitions	.838	.834
4. Weak Cognitions	.702	.737
5. Suicide Cognitions	.741	.764

**Figure 1.**
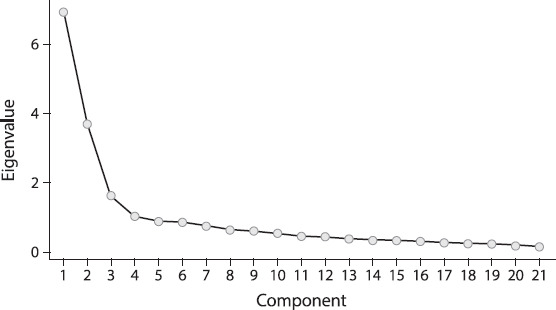
Scree Plot of Components of the NSSI-SCS Scale

The obtained results indicate that the original theoretical five-factor structure of the NSSI-SCS was not adequately replicated in the Russian-speaking sample, necessitating a revision of the scale’s factor structure for this cultural context. Consequently, to investigate the questionnaire’s factorial structure, an exploratory factor analysis (EFA) using principal component analysis (PCA) and oblimin rotation was conducted. The most suitable model was a three-factor solution, which was determined ater the sequential removal of items with factor loadings below .5. Inspection of the scree plot conirmed the presence of three factors (Cattell’s criterion) (*Figure 1*).

The internal consistency of the three-factor NSSI-SCS model was assessed using Cronbach’s alpha. The coefficients for all three factors exceeded .80, indicating good to excellent reliability (*Table 6*). Based on the scree plot analysis and these strong reliability indices, the irst three components were retained for the inal model.

**Table 6:** Internal Consistency Indicators of the NSSI-SCS

Indicator	Cronbach’s α	McDonald’s ω
Factor 1	.883	.889
Factor 2	.873	.875
Factor 3	.835	.84
Scale	.876	.882

A standard pattern matrix was used to interpret the three factors (*Table 7*). The irst factor was interpreted as “Hopelessness”, relecting cognitions that NSSI scars lead to thoughts of weakness, hopelessness, and death. The second factor, designated as “Shame”, encapsulated the belief that scars cause embarrassment and a fear of negative social evaluation. The third factor was labeled “Inner Strength”, representing the perception of scars as demonstrations of emotional and physical resilience and a source of personal pride. A comparison with the original NSSI-SCS revealed notable structural differences. The “Hopelessness” factor comprised a combination of items: four from the original “Suicide Cognitions” subscale, two from “Weak Cognitions”, and one from “Reminder Cognitions”. The “Shame” factor primarily reproduced the original Social Cognitions subscale. Th e Inner Strength factor closely mirrored the original “Positive Cognitions” subscale, with the exception of a single cross-loading item (Item 25) from the “Suicide Cognitions” subscale.

**Table 7:** Factor Loadings of NSSI-SCS Statements

NSSI-SCS Statement	Factor Loading	Uniqueness
Factor 1 (Hopelessness)		
19. My scars make me feel like I could kill myself if I wanted to.	.83	.396
22. My scars make me feel like I can’t change anything, like I’m stuck.	.8	.302
21. My scars make me feel like I can’t handle the future.	.793	.305
17. My scars make me feel like I want to kill myself.	.67	.511
6. My scar(s) remind me that I am weak.	.669	.45
15. My scars make me think about my failures and mistakes.	.638	.364
26. My scars make me feel less afraid of dying.	.62	.506
14. My scars make me feel hopeless.	.568	.336
9. My scars remind me of stressful things that happened to me in the past.	.509	.607
Factor 2 (Shame)		
10. My scars make me embarrassed in front of other people.	.861	.258
23. I think that people judge me because of my scars.	.762	.352
24. I think that I would have better relationships if I didn’t have any scars.	.757	.422
8. My scars make me feel shame.	.755	.43
16. I think that people stare at my scars.	.723	.447
18. My scars make me feel unattractive.	.711	.352
Factor 3 (Inner Strength)		
11. My scars make me feel tough, like I can get through anything.	.842	.29
1. My scar(s) represent how strong I am emotionally.	.796	.353
5. My scars make me feel proud that I got through a very tough time.	.793	.371
20. My scars make me feel hopeful about my future.	.684	.538
25. I think that my scars make me unique.	.657	.559
2. My scar(s) represent how strong I am physically.	.645	.581

The following items from the original scale were excluded from the final three-factor model due to insufficient factor loadings (all below the .5 threshold) and are presented in *Table 8*.

**Table 8:** Factor Loadings of Items Excluded from the Three-Factor NSSI-SCS Model

Factor Loadings				
NSSI-SCS Statement	Factor			Uniqueness
	1	2	3	
3. My scars bring back memories of things that I don’t want to remember	.13	.49	.204	.648
4. My scars make me think about how weak I used to be	.17	.38	.152	.742
7. My scars make me feel afraid	.332	.327	.09	.663
12. My scars make me feel like I am weak	.469	.312	-.23	.468
13. I think about my scars.	.444	.265	.12	.597

The three extracted factors collectively account for 58.4% of the total variance, which is considered satisfactory for clinical psychology and psychometric questionnaires (see *Table 9*). Th is figure exceeds the minimum threshold of 50% for valid psychological scales, indicating that the three-factor model adequately captures the primary patterns of inter-item relationships within the instrument.

**Table 9:** Variance Explained by the Three-Factor NSSI-SCS Structure

Factor	Sum of Squared Loadings (SS)	% of Variance	Cumulative %
1	4.57	21.8	21.8
2	4.06	19.3	41.1
3	3.64	17.3	58.4

A comparison of the original ive-factor model (see Table 4) and the three-factor model (see *Table 10*) using goodness-of-fit indices revealed that the three-factor model is statistically more justified for the obtained empirical data from the Russian-speaking sample.

**Table 10:** Goodness-of-Fit Indices for the Three-Factor NSSI-SCS Model

χ^2^ (Chi-square)		df (Degrees of freedom)		p-value	
636		186		< .001	
				RMSEA 90% CI	
**CFI**	**TLI**	**SRMR**	**RMSEA**	**Lower Bound**	**Upper Bound**
.826	.803	.0855	.101	.0927	.11

The three-factor model demonstrates a better normed chi-square (x^2^/df) ratio (636/186 = 3.42) compared to the five-factor model (817/220 = 3.71). Although both values exceed the acceptable threshold of 2, the difference indicates a superior fit for the three-factor solution. Furthermore, the CFI for the three-factor model (.826) is substantially higher than that of the five-factor model (.781) and approaches the acceptability threshold (> .90). Th e TLI for the three-factor model (.803) meets the marginal “tolerance” threshold of .80, unlike the five-factor model (TLI = .748). A key improvement is observed in the RMSEA value: .107 for the ive-factor model indicates a poor it, while .086 for the three-factor model is considered acceptable. However, the SRMR values for both models (.100 for the five-factor and .101 for the three-factor) exceed the strict threshold of .08, suggesting residual discrepancies remain in both conigurations. Overall, on all key indices except the SRMR, the three-factor model demonstrates a statistically more adequate it to the data.

A direct comparison using the Chi-Square Diference Test yielded a statistically significant result (Δχ^2^ = 181, Adf = 34, p < .001). This finding confirms that the three-factor model provides a statistically superior fit to the five-factor model, as it achieved a signiicant improvement in model it while utilizing fewer parameters.

**Figure 2.**
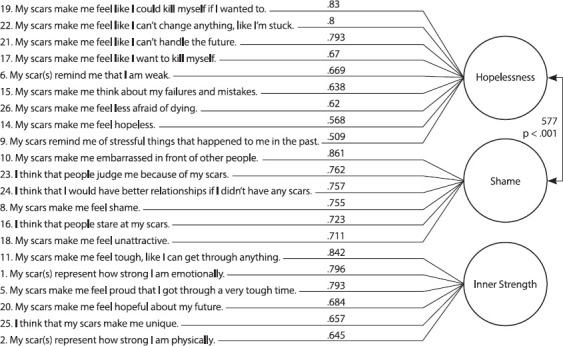
Intercorrelations between the Factors of the NSSI-SCS

Correlation analysis (Spearman’s ρ) revealed a substantial positive intercor-relation between the “Hopelessness” (F1) and “Shame” (F2) subscales (ρ = .577, p < .001). In contrast, the “Inner Strength” (F3) subscale demonstrated no significant correlation with either F1 or F2. This supports its conceptual independence, as it appears to tap into a distinct construct related to perceptions of resilience and personal strength derived from scars. Conversely, the “Hopelessness” and “Shame” subscales collectively relect the negative aspects of cognitive and emotional scar appraisal (*Figure 2*).

### Data on the Convergent and Discriminant Validity of the Non-Suicidal Self-Injury Scar Cognition Scale (NSSI-SCS)

Analysis of bivariate correlations (Spearman’s ρ) revealed a pattern of statistically significant relationships between the NSSI-SCS factors and the ISAS scales (see *Table 5*). It is important to note that while statistically significant due to the sample size, several of the observed correlation coefficients were modest in magnitude (ρ < .2). Nevertheless, their specific pattern contributes to the understanding of the scale’s construct validity. Furthermore, the “Hopelessness” subscale score was signiicantly correlated with a broad range of self-injury functions (ISAS), including Affect Regulation, Self-Punishment, Anti-Dissociation, Anti-Suicide, Interpersonal Boundaries, Self-Care, Sensation Seeking, Toughness, Autonomy, and Marking Distress. The “Shame” sub-scale showed a more speciic pattern of correlations, being signiicantly associated with Self-Punishment, Anti-Suicide, Interpersonal Boundaries, and Revenge (*Table 11* ). The “Inner Strength” score showed significant, though weak, positive correlations with a broad range of self-injury functions, including afect regulation, anti-dissociation, interpersonal boundaries, self-care, sensation seeking, toughness, autonomy, and marking distress (ρ values ranging from .136 to .362).

**Table 11:** Correlations (Spearman’s ρ) of NSSI-SCS factors with the Inventory of Statements about Self-Injury (ISAS)

Functions of Self-Injury (ISAS)	Factor 1 “Hopelessness”	Factor 2 “Shame”	Factor 3 “Inner Strength”
Affect regulation	.214***	.087	.185**
Self-punishment	.432***	.284***	.069
Anti-dissociation	.185**	-.01	.157*
Anti-suicide	.347***	.19**	.174**
Interpersonal boundaries	.239***	.184**	.237***
Self-care	.287***	.091	.271***
Sensation-seeking	.144*	.081	.351***
Peer-bonding	.071	.05	.136*
Interpersonal influence	.122	.079	.28***
Toughness	.217***	.081	.283***
Revenge	.118	.16*	.172**
Autonomy	.158*	.056	.222***
Marking distress	.289***	.105	.362***

Note.*p < .05, **p < .01, ***p < .001.

Analysis of discriminant validity established that the “Hopelessness” and “Shame” subscales of the NSSI-SCS demonstrated significant positive correlations (Spearman’s ρ) with total scores on measures of social anxiety (Liebowitz Social Anxiety Scale, LSAS), rumination (Ruminative Responses Scale, RRS), body-image dysphoria (Situational Inventory of Body-Image Dysphoria, SIBID), and suicide ideation (Beck Scale for Suicide Ideation). In contrast, the “Inner Strength” subscale showed no signiicant associations with these clinical constructs (*Table 12*).

**Table 12:** Bivariate Correlations (Spearman’s ρ) Between the Factors of the NSSI-SCS and Total Scores on the LSAS (Liebowitz Social Anxiety Scale), RRS (Ruminative Responses Scale), SIBID (Situational Inventory of Body-Image Dysphoria), and the Beck Scale for Suicide Ideation

	“Hopelessness”	“Shame”	“Inner Strength”
Suicidal risk	.43***	.15*	-.084
Social anxiety	.396***	.365***	.035
Body dissatisfaction	.495***	.401***	.035
Rumination	.427***	.383***	.056

Note.*p < .05, *** p < .001.

To identify diferences in NSSI-SCS scores between patients with scars in dif ferent locations (visible vs. hidden), a non-parametric Mann-Whitney U test wa conducted (*Table 13*). The analysis revealed a statistically significant difference be tween the groups on the “Inner Strength” subscale (U = 2917, p < .001). Respondent with visible scars (M = 10.2, SD = 4.79) reported significantly higher scores on thi subscale compared to those with concealed scars (M = 8.29, SD = 3.79). This resu indicates that individuals with scars apparent to others are more likely to perceiv them as symbols of resilience, emotional strength, and overcoming adversity. In con trast, no statistically signiicant diferences were found between the groups on th “Hopelessness” (U = 3887, p = .344) and “Shame” (U = 3913, p = .380) subscales. Thi suggests that the negative cognitive appraisals of scars — such as feelings of hopeless ness, suicidality, and social shame — are prevalent regardless of their visibility.

**Table 13:** Differences in NSSI-SCS scores between patients with scars in different locations (visible v hidden) Mann-Whitney U-test

	Visible scars (n = 121)		Hidden scars (n = 70)		Assessment of the validity of differences		
	M	SD	M	SD	Mann-Whitney U-test	p	Cohen’s d
Inner Strength	10.2	4.79	8.29	3.79	2917	<.001	.311
Hopelessness	18	8.61	18.29	7.06	3887	.344	.082
Shame	11.9	5.91	12.27	5.23	3913	.38	.076

A comparative analysis was conducted between respondents scoring in the highest (Q4) and lowest (Q1) quartiles on each of the NSSI-SCS subscales using the Mann-Whitney U test. Inner Strength Subscale. Th e proportion of respondents in the high-scoring group (Q4) was 33.5% (n = 79). When compared to the low-scoring group (Q1, n = 80), the high-scoring group demonstrated significantly greater endorsement of the following NSSI functions: Affect Regulation (p = .004), Anti-Dissociation (p = .036), Anti-Suicidal (p = .009), Interpersonal Boundaries (p < .001), Self-Care (p < .001), Sensation-Seeking (p < .001), Interpersonal Influence (p < .001), Toughness (p < .001), Revenge (p = .006), Autonomy (p = .004), and Marking Distress (p < .001), Hopelessness Subscale. High scores on this subscale were observed in 31.8% of participants (Q4, n = 75). This group, compared to the low-scoring group (Q1, n = 49), exhibited significantly elevated scores on established clinical measures: the Beck Scale for Suicide Ideation (p < .001), the Liebowitz Social Anxiety Scale (p < .001), the Situational Inventory of Body-Image Dysphoria (p < .001), and the Ruminative Responses Scale (p < .001). Furthermore, they reported higher scores on several NSSI functions, including Affect Regulation (p < .005), Anti-Dissociation (p = .004), Anti-Suicidal (p < .001), Interpersonal Boundaries (p < .003), Self-Care (p < .001), Sensation-Seeking (p = .012), Toughness (p < .001), Autonomy (p = .028), and Marking Distress (p < .001). Shame Subscale. Th e high-scoring group (Q4) for this subscale comprised 35.6% of respondents (n = 84). Comparative analysis with the low-scoring group (Q1, n = 41) revealed that the Q4 group had significantly higher scores on measures of social anxiety (p < .001), body-image dysphoria (p < .001), and rumination (p < .001). They also endorsed significantly higher scores on specific NSSI functions: Self-Punishment (p < .001), Anti-Suicidal (p = .048), Interpersonal Boundaries (p = .007), Sensation-Seeking (p = .045), and Marking Distress (p = .015).

## Discussion

Th e Russian-language adaptation of the Non-Suicidal Self-Injury Scar Cognition Scale (NSSI-SCS) resulted in a psychometrically robust 21-item instrument, structured around a three-factor model that demonstrated superior it and validity compared to the original i ve-factor structure. All three subscales exhibited high internal consistency (Cronbach’s α > .80). This adaptation process involved the removal of five items from the original 26-item English version due to insuicient factor loadings (< .5) in the exploratory analysis. The final model consolidates the original four negative cognition subscales — “Reminder Cognitions,” “Social Cognitions,” “Weak Cognitions,” and “Suicide Cognitions” — into two broader, clinically salient factors: 1. Hopelessness, which integrates cognitions related to suicidal ideation, personal weakness, and distressing reminders of past experiences. 2. Shame, which captures experiences of social stigmatization and embarrassment.

The original “Positive Cognitions” subscale was retained and conceptually reined as “Inner Strength,” relecting the perception of scars as a symbol of personal resilience and endurance. This three-factor structure is consistent with established theoretical models of scar perception following NSSI (*e.g.*, Bachtelle & Pepper, 2015; Lewis & Mehrabkhani, 2016).

In this way this study operationalizes a set of latent constructs reflecting the cognitive appraisal of scars following NSSI. The construct ‘Inner Strength’ is theoretically defined as the perception of scars as symbols of resilience, overcoming adversity, and emotional endurance. The construct ‘Shame’ is defined as a cognitive-affective response encompassing the anticipation of social judgment and feelings of body rejection. The construct ‘Hopelessness’ amalgamates cognitions linking scars to feelings of doom, suicidal ideation, and an inability to change.

The study found that the “Inner Strength” indicator is a specific construct associated with the functionality of non-suicidal self-injurious behavior. Similar findings were reported in the study by T. Burke (Burke et al., 2017). Its lack of association with measures of psychological distress (LSAS, RRS, SIBID, BSSI) suggest its relative independence. A person may experience high social anxiety or rumination without associating these states with their scars The identified pattern of weak-to-moderate correlations between the “Inner Strength” factor and the functions of self-injury suggests its potential role in relective aspects of coping with the consequences of non-suicidal self-injury. Th e “Inner Strength” factor appears to tap into a parallel cognitive process where scars are appraised through a lens of resilience and survival. It is important to acknowledge that the correlations with ISAS functions, while statistically significant, were generally modest (see *Table 5*). This indicates that viewing scars as a symbol of inner strength is a distinct, though not predominant, cognitive pathway that can coexist with both adaptive and maladap-tive coping mechanisms which aligns with indings from other studies (Kendall et al., 2021; Linington, 2024).

The “Inner Strength” factor identified in our study finds a compelling parallel in contemporary clinical research (Sripunya et al., 2024) identified “inner strengths” — conceptualized as resilience and self-compassion — as a key mediator attenuating the relationship between emotional distress and self-injurious behaviors. While their construct is measured more broadly, the resonance with our indings suggests that perceiving scars as symbols of resilience may tap into a similar protective psychological resource, rather than being merely a cognitive reappraisal. Th erefore, our findings, consistent with the broader literature on psychology (e.g., Zhao et al., 2023), suggest that therapeutic approaches could beneit from investigating a shit in focus from exclusively mitigating negative symptoms toward the deliberate cultivation of positive psychological resources.

Non-suicidal self-injury has pronounced negative long-term consequences, leading to a vicious cycle of distress (Burke et al., 2017). Th e relationship between the indicator of Negative appraisal of scars (including the indicators “Hopelessness” and “Shame”, affecting about one-third of the sample) and worsening of psychopathologi-cal symptoms: increased suicidal ideation, greater social anxiety, body dissatisfaction, and rumination, which is consistent with previous research (Burke et al., 2020; Glenn, 2024).

However, the modest magnitude of several correlations (e.g., ρ ~ .2) suggests that, while statistically signiicant and theoretically meaningful, the shared variance between these constructs is limited. This indicates that the NSSI-SCS factors capture unique aspects of scar-related cognition not fully explained by general measures of suicidality, anxiety, rumination, or body image.

Our analysis of scar visibility using the Mann-Whitney U test revealed a clinically signiicant dissociation in cognitive appraisals. While no diferences emerged on the “Hopelessness” (U = 3887, p = .344) and “Shame” (U = 3913, p = .380) sub-scales — suggesting the pervasive nature of negative scar-related cognitions regardless of visibility —individuals with visible scars reported significantly higher “Inner Strength” scores (U = 2917, p < .001). Th is pattern aligns with previous qualitative research (Chandler, 2014; Lewis & Mehrabkhani, 2016). The persistent shame and hopelessness across both groups corroborates indings by Burke et al. (2020) that concealment strategies may not mitigate underlying psychological distress. However, the enhanced “Inner Strength” appraisals in the visible scars group suggest that public visibility might, for some individuals, facilitate a narrative transformation process wherein scars become integrated into identity as markers of resilience rather than merely concealed sources of shame.

The results of the Russian adaptation, which differ from the English version, largely relect cultural diferences between Western and Eastern methodologies (Myung et al., 2024). In Western cultures (especially individualistic societies like the United States), more attention is paid to individual experiences. In Russian-speaking culture, which is more aligned with collectivist models, “stigma and shame” play a greater role. Whereas Western models of NSSI often frame the behavior through the lens of emotional regulation, Eastern cultures place more emphasis on the social context, embarrassment, and stigma. This is consistent with the emergence of “shame” as a separate factor in the Russian version. This cultural specificity is supported by empirical evidence from other studies, for instance, research by Thompson & Bhugra (2000). Another study (Gholamrezaei et al., 2017) authors highlight the heightened salience of interpersonal functions of NSSI in collectivist contexts, as well as the substantial role of culturally-bound stress.

Furthermore, research by Chen et al. (2021) demonstrates the considerable influence of the social context and collectivistic values on the pronounced experience of shame and guilt associated with self-injurious behaviors.

The three-factor structure of the Russian-language version of the NSSI-SCS obtained in our study finds empirical parallels in cross-cultural validation studies of other clinical instruments. Speciically, the validation of the Serbian version of the Clinical Assessment Interview for Negative Symptoms (CAINS) revealed a three-factor structure differing from the original two-factor model (Ristic et al., 2020). Across various cultural contexts have repeatedly demonstrated diferences in factor structure of different clinical instruments from the original model, without precluding the valid use of the adapted versions. Further supporting the methodological approach of identifying a culturally specific factor structure, our findings align with adaptation studies of other well-established psychological instruments in Russia. The large-scale validation of Budner’s Tolerance-Intolerance of Ambiguity Scale (Kornilova & Chumakova, 2014) conirmed a stable two-factor model distinguishing Tolerance for Uncertainty (TU) and Intolerance for Uncertainty (IU) as separate constructs, rather than a unidimensional one.

## Limitations and prospects

Among the limitations of the study, it is important to note the characteristics of the surveyed groups — most of the sample consisted of women. The gender imbalance in the sample, characterized by a predominance of women (74.4%), relects established epidemiological patterns of NSSI prevalence, which consistently show a higher prevalence of this behavior in the female population. However, we acknowledge that the underrepresentation of men limits the ability to extrapolate the indings to the male population and consider this an important direction for future research.

Another limitation is the lack of an assessment of test-retest reliability. Furthermore, the interpretation of some correlational findings, particularly for the “Inner Strength” subscale, should be tempered by the recognition that several statistically signiicant correlations were weak in magnitude (ρ < .2), their practical and clinical signiicance may be limited. Further clariication is needed regarding the concept of “Inner Strength” as a speciic attitude toward one’s own scars. While this indicator relects a perspective contrary to the negative appraisal of scars, it does not show inverse correlations with measures of general psychological distress.

The prospect for further research is the practical application of the adapted version. Studies based on this can be used to develop recommendations for the prevention of NSSI relapses and to make decisions about cosmetic surgery and body modiications.

## Conclusions

This study on the validation of the Russian-language version of the Nonsuicidal Self-Injury Scar Cognition Scale (NSSI-SCS) highlights the key role of subjective scar perception in the psychological well-being of individuals with a history of self-injurious behavior.

The findings of the present study provide comprehensive support for the construct validity of the Russian-language NSSI-SCS. Unlike the original five-factor model, validation with a Russian-speaking sample revealed a conceptually grounded three-factor structure: 1) “Hopelessness” — integrates internal distress (suicidal ideation, feelings of weakness), reflecting global negative self-perception. 2) “Shame” — focuses on social distress (stigmatization, embarrassment), which holds particular conceptual signiicance in cultures with collectivistic characteristics. 3) “Inner Strength” — represents an independent positive construct symbolizing resilience and overcoming adversity.

Structural validity, established via EFA and CFA, confirmed a three-factor model consistent with theoretical conceptions of the cognitive appraisal of scars. Convergent validity were largely supported by predictable moderate-to-strong associations of the ‘Hopelessness’ and ‘Shame’ scales with relevant measures. Discriminant validity of the ‘Inner Strength’ scale was also supported, underscoring its uniqueness as a construct distinct from general negative afect. Finally, conirmed validity based on the diferences between groups with visible and invisible scar locations adds robust evidence in favor of the measure’s validity.

The three-factor model represents a culturally adapted and conceptually valid framework that more accurately captures the key dimensions of scar perception within Russian-speaking populations. This model provides a more parsimonious yet profound understanding of the cognitive landscape associated with the consequences of non-suicidal self-injury in this specific cultural context.
